# Involvement of Monocyte Subsets in the Immunopathology of Giant Cell Arteritis

**DOI:** 10.1038/s41598-017-06826-4

**Published:** 2017-07-26

**Authors:** Yannick van Sleen, Qi Wang, Kornelis S. M. van der Geest, Johanna Westra, Wayel H. Abdulahad, Peter Heeringa, Annemieke M. H. Boots, Elisabeth Brouwer

**Affiliations:** 1 Department of Rheumatology and Clinical Immunology, University of Groningen, University Medical Center Groningen, Groningen, The Netherlands; 2 Department of Pathology and Medical Biology, University of Groningen, University Medical Center Groningen, Groningen, The Netherlands

## Abstract

Monocytes/macrophages are critical in systemic and local inflammation in giant cell arteritis (GCA) and possibly in clinically overlapping polymyalgia rheumatica (PMR). Therefore, we aimed to understand the contribution of monocyte subsets and the CX3CR1-CX3CL1 and CCR2-CCL2 migratory pathways, to the pathology of GCA. Peripheral blood monocytes were enumerated in samples from newly-diagnosed, untreated GCA and PMR patients and after prednisone-induced remission. The distribution of classical (CD14^bright^CD16^neg^) and the more pro-inflammatory, intermediate (CD14^bright^CD16+) and non-classical (CD14^dim^CD16+) monocyte subsets was analysed by flow cytometry. The phenotype of macrophages in temporal artery biopsies (TABs) from GCA patients was studied by immunohistochemistry and immunofluorescence. A clear monocytosis was seen in newly diagnosed GCA and PMR patients caused by elevated numbers of classical monocytes. Prednisone treatment suppressed numbers of non-classical monocytes. Both chemokine CX3CL1 and CCL2 were highly expressed in the TAB. Most macrophages in the TAB of GCA patients expressed non-classical monocyte markers CD16 and CX3CR1 whereas co-localisation of CD16 with classical monocyte marker CCR2 was infrequent. In conclusion, we report an altered distribution of monocyte subsets in both GCA and PMR patients. The majority of macrophages in TABs of GCA patients were CD68 + CD16 + CX3CR1 + CCR2− and thereby resembled the phenotype of non-classical monocytes.

## Introduction

Giant cell arteritis (GCA) is an immune mediated vasculitis characterized by granulomatous infiltrates in the vascular wall of medium and large arteries causing vascular occlusion leading to blindness or stroke. GCA is not solely a ‘’headache disease” (cranial GCA (C-GCA)) but can present with systemic vessel inflammation (large vessel GCA (LV-GCA)). Both C-GCA and LV-GCA patients can have signs and symptoms of polymyalgia rheumatica (PMR), which is characterized by pain and stiffness of both shoulders and hips and by systemic inflammation. PMR is observed in 50% of GCA patients and 15% of patients with PMR may develop GCA when left untreated. As GCA and PMR develop in persons over 50 years of age, with a median age of 70 at onset, it has been suggested that ageing-associated changes of the immune system may be involved^1–3^. Glucocorticoid treatment is currently the first choice for clinical management of GCA and PMR, but long-term glucocorticoid treatment is associated with severe side effects^4^. An improved understanding of the immunopathogenesis of GCA and PMR may eventually lead to highly needed alternative treatment options for GCA and PMR patients.

The immunopathogenesis of both GCA and PMR is not yet well understood. There is consensus, however, that GCA pathology is initiated by local dendritic cell activation followed by infiltration of the vessel wall by CD4+ T-cells and monocytes/macrophages via the vaso vasorum^5^. Within the vessel wall, migrated monocytes/macrophages produce pro-inflammatory cytokines and matrix metalloproteases causing severe vascular damage. Monocytes, the precursors of tissue infiltrating macrophages, are phagocytes generated in the bone marrow from which they are released into the bloodstream where they circulate for several days^6^. Three monocyte subsets can be distinguished by phenotypic and functional characteristics: classical monocytes (CD14^bright^CD16^neg^), intermediate monocytes (CD14^bright^CD16+) and non-classical monocytes (CD14^dim^CD16+)^7^. CD14^bright^CD16^neg^ classical monocytes represent the most abundant subset in the peripheral blood whereas the pro-inflammatory CD16+ subsets (both intermediate and non-classical) are less frequent^8^. CD16+ monocytes are the more mature cells compared to the classical monocytes; a developmental relationship has been established, and their numbers increase with age^9^. Importantly, increased proportions of CD16+ monocytes have been associated with numerous vascular and inflammatory diseases like RA^10^, sarcoidosis^11, 12^, SLE^8^ and ANCA-associated vasculitis^13, 14^.

To study the contribution of monocytes/macrophages to the immunopathogenesis of GCA, it is crucial to understand the monocyte subsets as precursors of the tissue macrophages and their chemokine directed migration in this disease. Tissue migration of different monocyte subsets is determined by differential expression of chemokine receptors^15^. Classical monocytes show a marked CCR2^bright^CX3CR1^dim^ expression whereas non-classical monocytes show CCR2^neg^CX3CR1^bright^ expression^16^. Also, CD16+ monocytes show an increased capacity to adhere to endothelial cells and thereby more readily migrate across the endothelium when compared to CD16^neg^ monocytes^17, 18^. Migration of CD16+ monocytes is guided by fractalkine (CX3CL1) – CX3CR1 interaction and inhibition of this interaction reduces transmigration^19, 20^.

So far, the distribution of the three monocyte subsets in GCA and PMR patients has not been studied. Moreover, as CD16+ monocytes are pro-inflammatory and increase with age, we hypothesized that these monocytes preferentially migrate to the vascular wall and contribute to GCA pathogenesis. We therefore studied monocyte subset distribution in newly diagnosed GCA and PMR patients and effects of prednisone treatment on these subsets. Next, we assessed whether CD16 was expressed by macrophages in temporal artery biopsies (TABs) of GCA patients. Lastly, we investigated expression of defined chemokine receptors and their ligands in peripheral blood of GCA and PMR patients and in temporal arteries of GCA patients.

## Results

### Monocyte counts are elevated in newly diagnosed GCA and PMR patients

**Figure 1:**
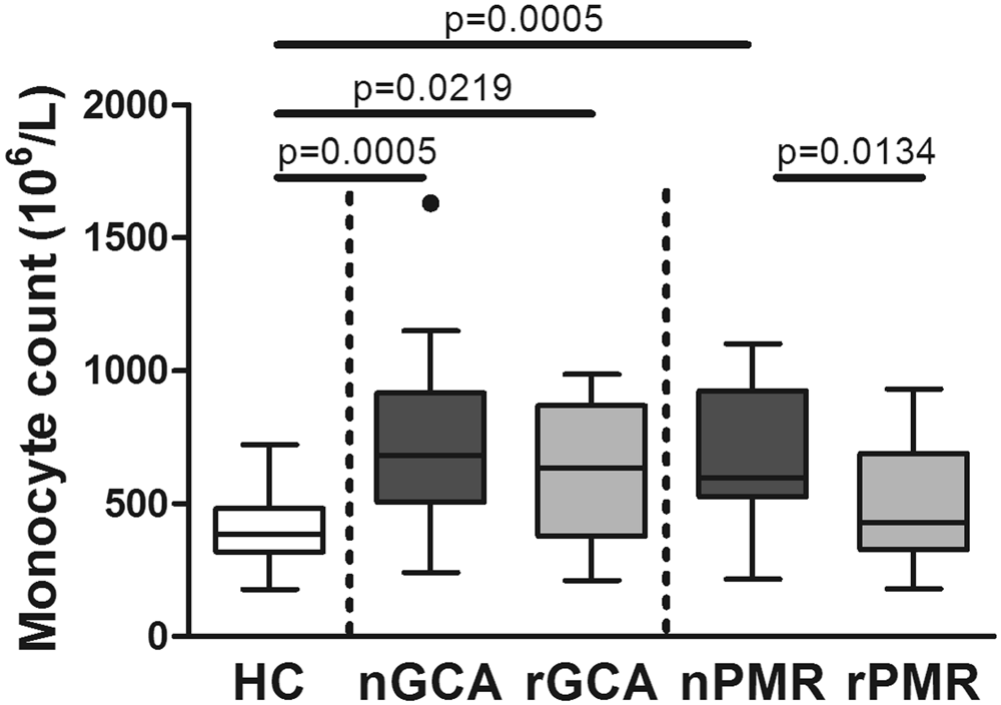
Total monocyte counts are elevated in newly diagnosed GCA and PMR patients. Absolute numbers of monocytes in freshly drawn whole blood obtained from healthy controls (HC, n = 20), newly-diagnosed patients with GCA (nGCA; n = 21), PMR (nPMR; n = 19) and in follow-up samples from patients in remission after 3 months of treatment (rGCA; n = 14, rPMR; n = 15). Data are expressed as Tukey box and whisker plots. The bottom and top of the box represent the first and third quartiles, and the band inside the box represents the second quartile (the median). The whiskers represent the 1.5 interquartile range (IQR) of the lower and the upper quartiles. The outlier is plotted as a dot. The Mann-Whitney U test was used to compare each patient group with HC. Paired samples (e.g. nGCA vs rGCA and nPMR vs rPMR) were compared with the Wilcoxon signed rank test. P-values of less than 0.05 (2-tailed) were considered statistically significant. P values are indicated in the graph.

Numbers of circulating monocytes as determined in a standard hematology counter were higher in newly diagnosed GCA (nGCA) and newly diagnosed PMR (nPMR) patients compared to HC (Fig. 1 and Tables 1 and S1). In addition, we assessed the effects of glucocorticoids after 3 months on treatment. Treatment led to normalization of monocyte numbers in remission PMR patients (rPMR) but this was not the case for monocyte numbers in remission GCA (rGCA) patients.

**Table 1:** Baseline characteristics of patients and controls in the peripheral blood study.

	nGCA (N = 22)	nPMR (N = 20)	HC (N = 24)
Age (yr); Median (range)	71 (52–81)	75 (54–84)	72 (53–83)
Females (%)	73	70	75
GCA diagnosis: PET-CT/TAB/PET-CT + TAB	11/5/6	NA	NA
PMR diagnosis: PET-CT/Chuang/PET-CT + Chuang	1/0/4	1/6/13	NA
Leukocytes (10^9^/L); Median (Range)	9.2 (5.0–18.4) *p* < *0*.*0001*	8.7 (4.5–14.4) *p* = *0*.*0011*	6.0 (4.2–9.6)
Hb (mmol/l); Median (Range)	7.0 (5.5–8.5) *p* < *0*.*0001*	7,5 (5.6–9.3) *p* < *0*.*0001*	8,7 (7.2–9.9)
ESR (mm/h); Median (Range)	65 (31–118) *p* < *0*.*0001*	52 (30–124) *p* < *0*.*0001*	12 (2–30)
CRP (mg/l); Median (Range)	47 (11–138) *p* < *0*.*0001*	44 (7–186) *p* < *0*.*0001*	2 (2–5)

Characteristics of newly diagnosed glucocorticoid/DMARD-free GCA (nGCA) and PMR patients (nPMR) and of healthy controls (HC). Five out of 22 GCA patients also had PMR. In 14 out of 20 PMR patients LV-GCA was excluded based on PET-CT. Importantly, the diagnosis of PMR did not change into GCA during a minimal follow-up of 6 months. The Kruskal-Wallis test was performed to compare data among the three study groups. The Mann-Whitney U test was used to compare each patient group with HC. P-values of less than 0.05 (2-tailed) were considered statistically significant. Yr = years. PET-CT = positron emission tomography-computed tomography. TAB = temporal artery biopsy. Hb = haemoglobin, ESR = erythrocyte sedimentation rate. CRP = C-reactive protein. NA = not applicable.

### Altered distribution of circulating monocyte subsets in GCA and PMR

**Figure 2:**
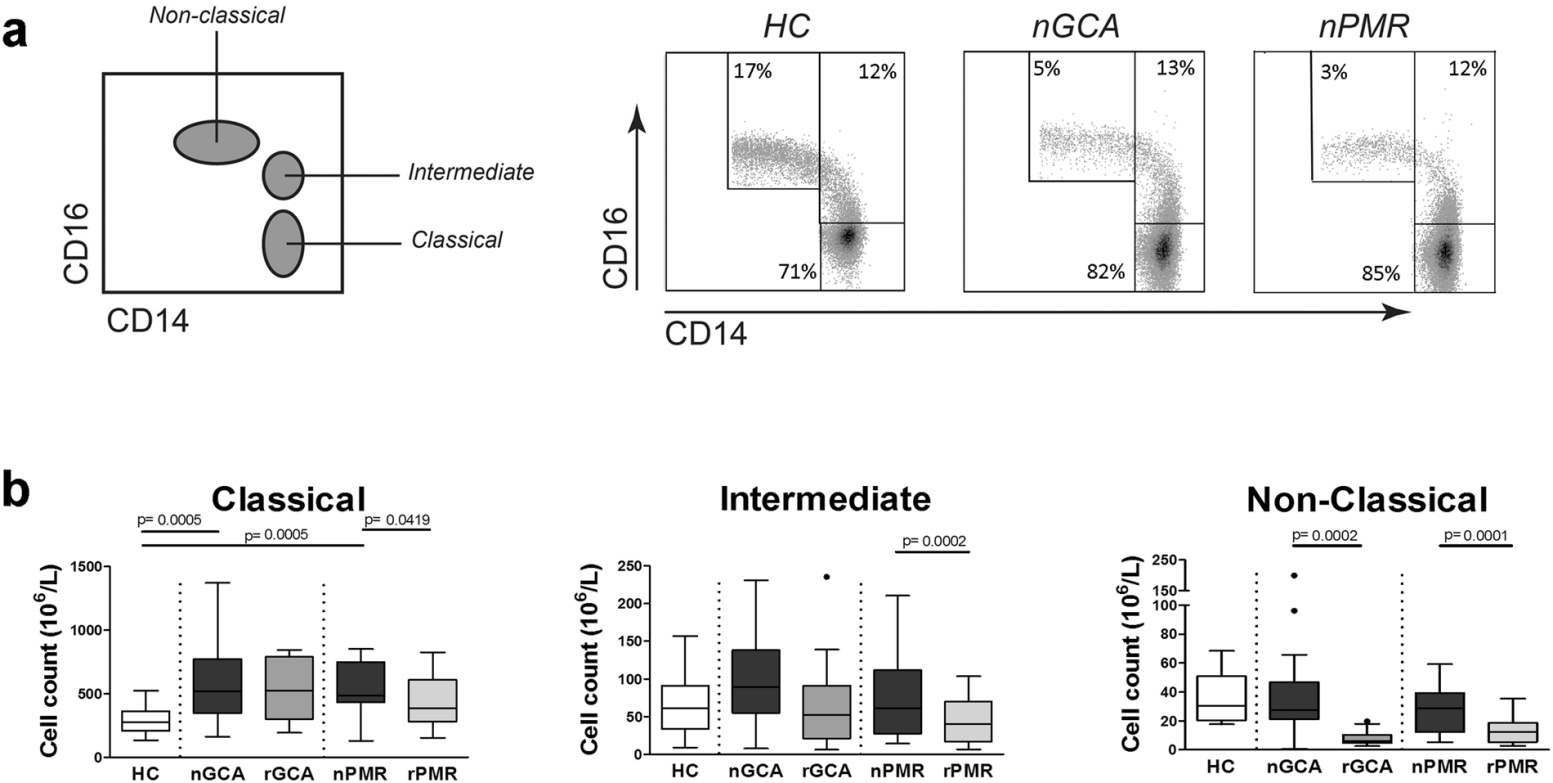
Altered distribution of monocyte subsets in GCA and PMR. (**a**) Flow cytometry gating strategy based on CD14 and CD16 expression to distinguish classical (CD14^bright^CD16^neg^), intermediate (CD14^bright^CD16^+^) and non-classical (CD14^dim^CD16^+^) monocytes subsets (left panel). Flow cytometry dot plots showing representative samples (equal numbers of events) from a healthy control (HC), a newly diagnosed GCA patient (nGCA) and a newly diagnosed PMR patient (nPMR) (right panel). (**b**) Absolute numbers of classical, intermediate, and non-classical monocytes in HC (HC, n = 20), newly-diagnosed patients with GCA (nGCA; n = 21) and PMR (nPMR; n = 19) and in the follow-up samples of GCA (rGCA; n = 14) and PMR (rPMR; n = 15) patients in remission after 3 months of glucocorticoid treatment. Data are expressed as Tukey box and whisker plots. The bottom and top of the box represent the first and third quartiles, and the band inside the box represents the second quartile (the median). The whiskers represent the 1.5 IQR of the lower and the upper quartiles. Outliers are plotted as dots. The Kruskal-Wallis test was performed to compare data among study groups. The Mann-Whitney U test was used to compare each patient group with HC. Paired samples were compared with the Wilcoxon signed rank test. P-values of less than 0.05 (2-tailed) were considered statistically significant.

As a clear monocytosis was observed in nGCA/nPMR, we next analysed the distribution of the three different monocyte populations defined by CD14 and CD16 expression (Fig. 2a). Our analysis revealed numerical increases of classical monocytes in nGCA and nPMR patients when compared to HC, whereas numbers of intermediate and non-classical monocytes were largely comparable (Figs 2b and S1). The increase in classical monocytes led to proportional decreases of non-classical monocytes in both nGCA and nPMR patients, but did not alter intermediate type monocyte proportions (Figs 2a and S1). Glucocorticoid treatment reduced the numbers of all three monocyte subsets in rPMR patients (Fig. 2b). Interestingly, in rGCA patients, 3 months of glucocorticoid treatment reduced numbers of non-classical monocytes but had no effect on numbers of classical and intermediate monocytes. Thus, both nGCA and nPMR patients are characterized by higher numbers of classical monocytes leading to proportional reductions of non-classical monocytes. Remarkably, clinical remission in GCA patients was associated with a clear reduction of non-classical monocytes.

### CD16+ macrophages are readily detected in GCA TABs

**Figure 3:**
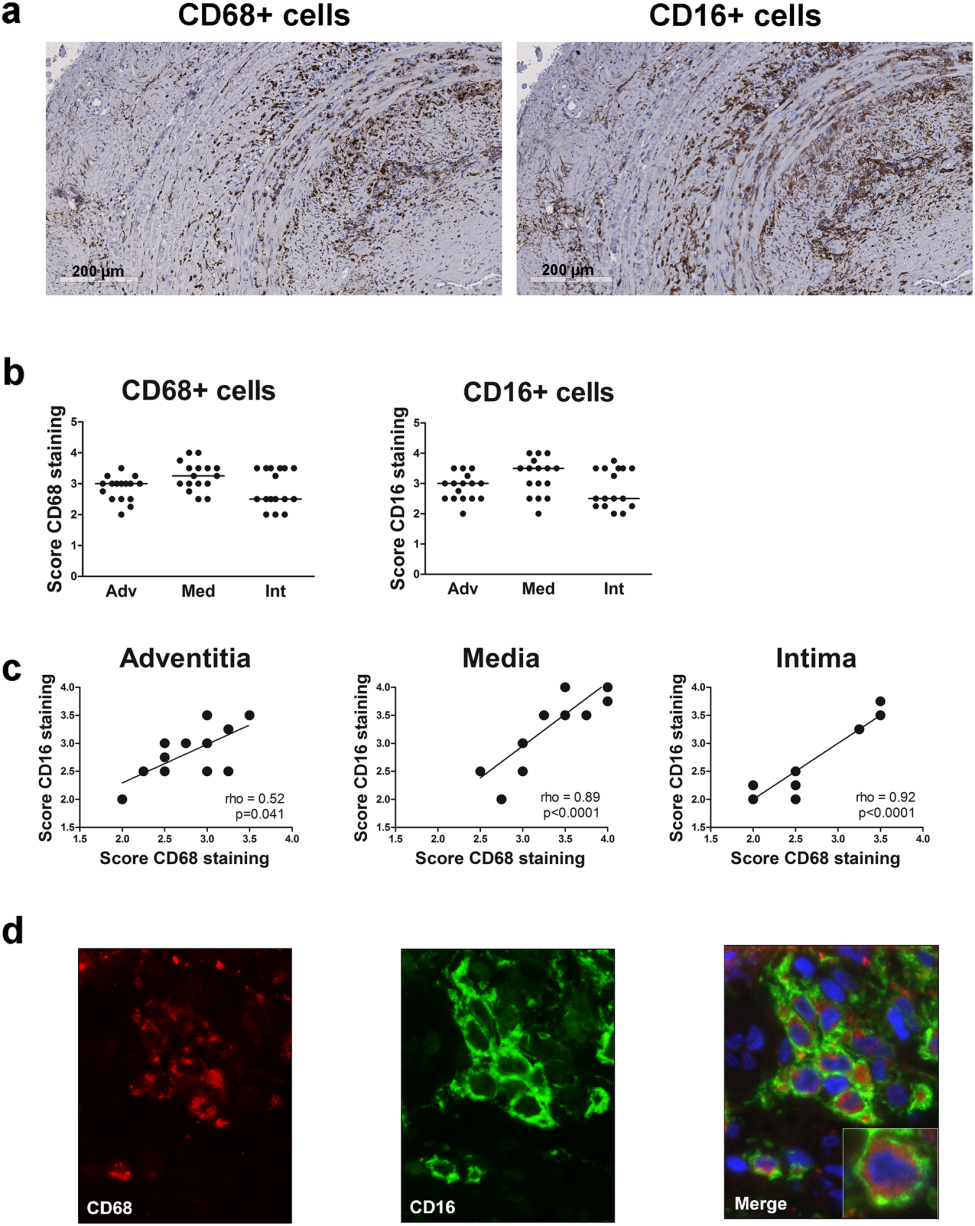
CD16+ cells co-localize with CD68+ macrophages in GCA temporal artery biopsies. (**a**) Immunohistochemical staining for CD68 and CD16 in a representative temporal artery biopsy (TAB) diagnostic of GCA. Note that consecutive sections of the same tissue were used in Figs 3 and 5. (**b**) Semi-quantitive mean scores of CD68+ cells and CD16+ cells in inflammatory areas of GCA TABs (n = 16). Scores are given for the adventitia (Adv), media (Med, infiltrating cells only) and intima (Int). Data are presented as scatter plots. The horizontal line indicates the median. (**c**) Positive correlation between CD16 and CD68 scores in intima, media and adventitia in inflammatory areas of GCA TABs (n = 16), as determined by Spearman’s rank correlation coefficient. Due to overlap in semi-quantitative scores not all 16 scores can be appreciated. (**d**) Single staining for CD68, CD16 and double staining for CD16 and CD68, respectively, from left to right in the inflammatory area of a TAB section. An example of a magnified merged picture is shown for clarity. Blue = DAPI staining of nuclei; Green (FITC) = CD16 expression, Red (AF555) = CD68 expression; a macrophage cytoplasmic granules marker.

As monocytes are recruited from the blood to peripheral sites of inflammation, where they differentiate into macrophages, we next investigated the phenotype of tissue macrophages in TABs with findings diagnostic of GCA and evaluated expression of CD16 and of macrophage marker CD68 (Figs 3a and S2 for isotype control stainings). CD68 and CD16 were both abundantly expressed within the infiltrates of the adventitia, media and intima layer of the vessel wall (Fig. 3b). CD16 expression was found to overlap with macrophage-rich areas as evidenced by CD68 expression. Indeed, scores of CD16+ and CD68+ cells in adventitia, media and intima were positively correlated (Fig. 3c). To rule out an involvement of NK cells expressing CD16, we stained for CD56 and found this marker to be virtually absent in the vessel wall. To further confirm the co-localization of CD68 and CD16, double immunofluorescence staining was performed. Indeed, within infiltrated regions, substantial co-localization of CD16 and CD68 was found, confirming that the majority of macrophages in the vascular wall express CD16 (Fig. 3d).

### Monocyte CCR2 and CX3CR1 expression in GCA and PMR patients

**Figure 4:**
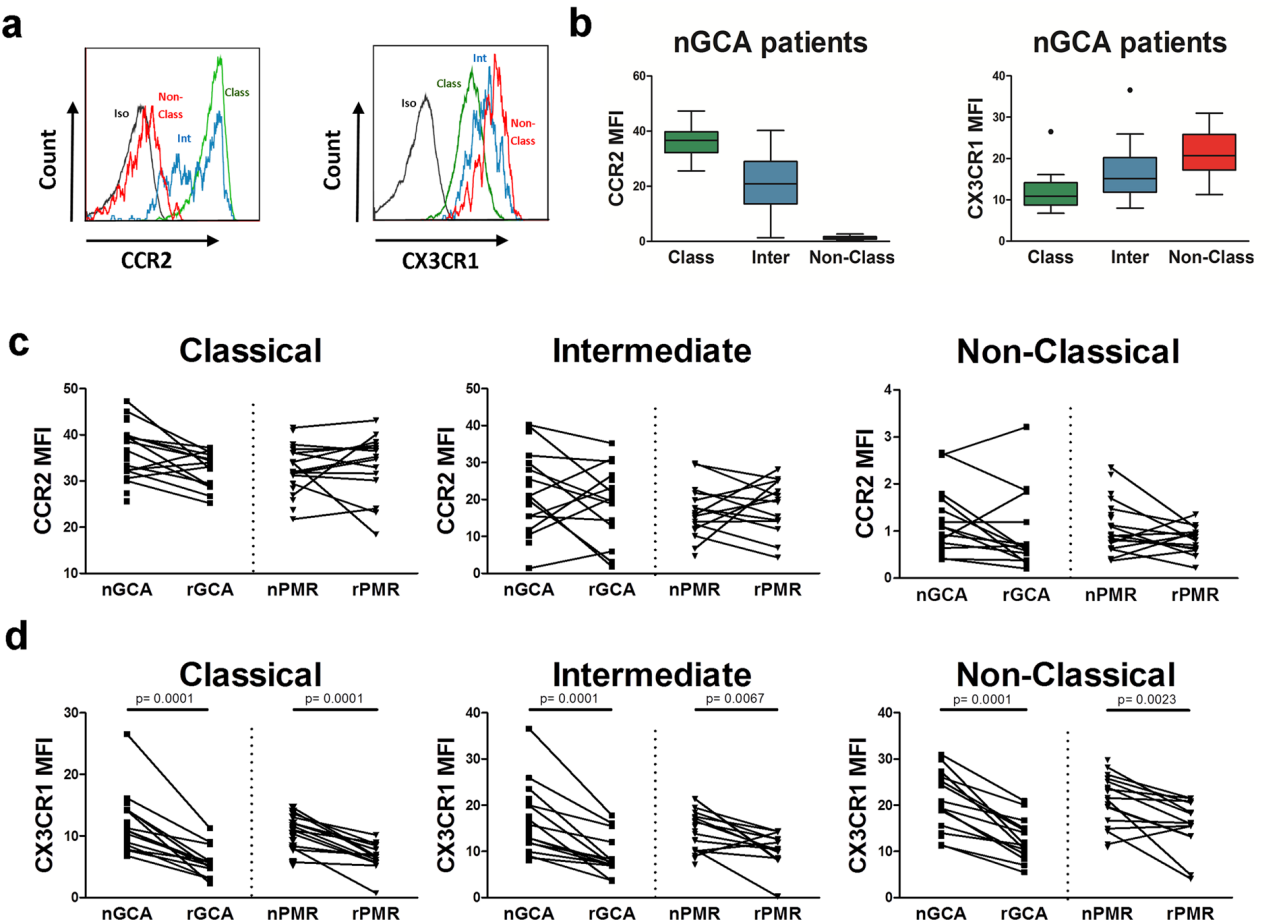
Expression of CCR2 and CX3CR1 by monocyte subsets in peripheral blood of GCA and PMR patients and effects of glucocorticoid treatment. (**a**) Representative flow cytometry dot plots of CCR2 and CX3CR1 by classical, intermediate and non-classical monocytes in a GCA patient. (**b**) Mean fluoresecence intensity (MFI) of CCR2 and CX3CR1 on classical (Class), intermediate (Inter) and non-classical (Non-Class) monocytes in newly diagnosed GCA patients (nGCA; n = 21). Data are expressed as Tukey box and whisker plots. The bottom and top of the box represent the first and third quartiles, and the band inside the box represents the second quartile (the median). The whiskers represent the 1.5 IQR of the lower and the upper quartiles. Outliers are plotted as dots. (**c**) Mean fluorescence intensity (MFI) of CCR2 and CX3CR1 (**d**) on classical, intermediate and non-classical monocytes in newly diagnosed GCA (nGCA; n = 21) and PMR (nPMR; n = 20) patients and in the follow-up samples of GCA (rGCA; n = 15) and PMR (rPMR; n = 14) patients in remission after 3 months of glucocorticoid treatment. Data are expressed as dot plots linking individual paired data. The Wilcoxon signed rank test was used to compare paired samples. P values are indicated in the graph.

Recruitment of monocytes to tissues is driven by specific chemokine and chemokine receptor interactions. As the three different monocyte subsets can also be distinguished by differences in their chemokine receptor expression profiles, we assessed expression of CCR2 and CX3CR1 on classical, intermediate and non-classical monocytes. In accordance with previous studies^21^, classical monocytes demonstrated a high per cell expression (Mean Fluorescence Intensity) of CCR2 with low expression of CX3CR1, while non-classical monocytes demonstrated high per cell expression of CX3CR1 and a lack of CCR2 expression (Fig. 4a,b). Following glucocorticoid treatment, CX3CR1 expression was down-modulated by all monocyte subsets in rGCA and rPMR patients. In contrast, CCR2 expression by monocyte subsets was not sensitive to glucocorticoid treatment (Fig. 4c,d).

### Systemic expression of CCR2 and CX3CR1 ligands in GCA and PMR patients

Next, we assessed if serum levels of the relevant chemokines were altered in the patient groups (Supplementary Table S2). Systemic levels of CCL2 and CCL11, the CCR2 receptor ligands, were lower in nGCA patients but normalized after glucocorticoid treatment. A similar pattern was observed in PMR patients but this did not reach statistical significance. In contrast, levels of both the CX3CR1 receptor ligands CX3CL1 (fractalkine) and CCL26 were not altered in GCA and PMR patients when compared to HC. Glucocorticoid treatment upregulated serum levels of CX3CL1 in rGCA patients.

Thus, our combined data show a down modulation of CX3CR1 expression by monocyte subsets and a concomitant increase of the soluble form of CX3CL1 in rGCA patients. Also, CCR2 expression by monocyte subsets was not altered in nGCA and nPMR patients when compared to HC (data not shown), but reduced levels of the CCR2 ligands were noted. The latter may be explained by high consumption/binding to increased numbers of circulating CCR2-positive monocytes in newly diagnosed GCA and PMR patients.

### Expression of CCR2, CX3CR1 and their ligands in GCA TABs

**Figure 5:**
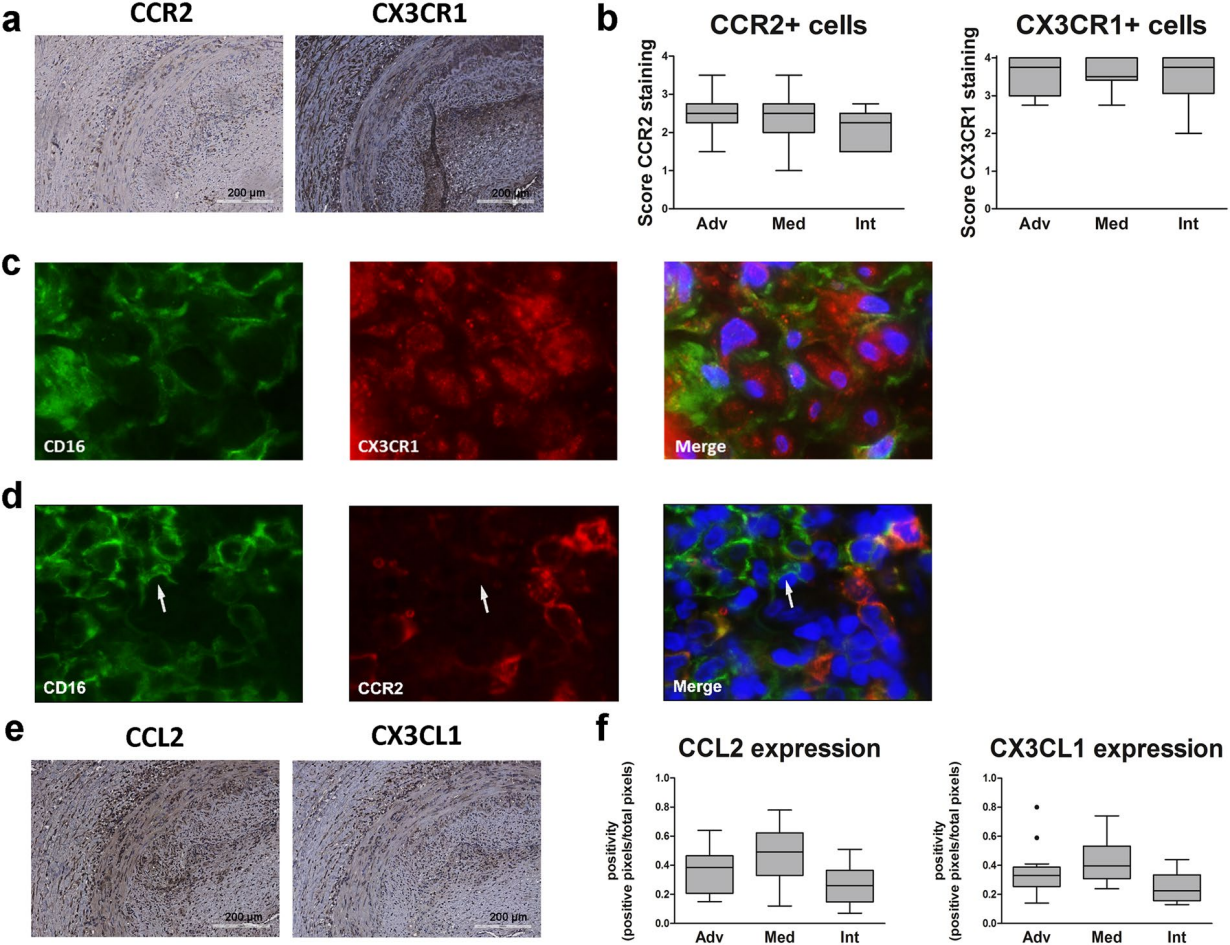
Expression of CCR2, CX3CR1 and their ligands in GCA temporal artery biopsies. (**a**) Immunohistochemical (IHC) staining for CCR2 and CX3CR1 in a representative temporal artery biopsy diagnostic of GCA. Note that consecutive sections of the same tissue were used in Figs 3 and 5. (**b**) Semi-quantitive score of CCR2-, CX3CR1-postive cells in the inflammatory area of GCA TAB (n = 15 and n = 16, respectively). Intensity of staining was not taken into account. Scores are given for the adventitia (Adv), media (Med, infiltrating cells only) and intima (Int). Data are presented as Tukey box and whisker plots. The bottom and top of the box represent the first and third quartiles, and the band inside the box represents the second quartile (the median). The whiskers represent the 1.5 IQR of the lower and the upper quartiles. (**c**) Co-localization of CD16 and CX3CR1 in TAB from GCA patients. Single staining for CD16, CX3CR1 and double staining for CD16 and CX3CR1, respectively from left to right in the inflammatory area of a TAB section. Blue = DAPI staining of nuclei; Green(FITC) = CD16 expression, Red(AF555) = CX3CR1 expression. (**d**) CCR2 and CD16 rarely co-localize in TAB from GCA patients. Single staining for CD16, CCR2 and double staining for CD16 and CCR2, respectively from left to right in the inflammatory area of a TAB section. Blue = DAPI staining of nuclei; Green(FITC) = CD16 expression, Red(AF555) = CCR2 expression. (**e**) IHC staining for CCL2, CX3CL1 in a representative TAB. (**f**) Quantification of staining intensity (positive pixels/total number of pixels) of CCL2 and CX3CL1 in the inflammatory vessel wall (n = 16 for both markers). Data are presented as Tukey box and whisker plots. The bottom and top of the box represent the first and third quartiles, and the band inside the box represents the second quartile (the median). The whiskers represent the 1.5 IQR of the lower and the upper quartiles. Outliers are plotted as dots.

Next, we investigated expression of CCR2, CX3CR1 and their ligands in TABs of GCA patients. In the inflamed vascular wall, high numbers (median score 3–4) of CX3CR1-positive cells were detected (Fig. 5a,b). CX3CR1-positive cells were clearly detected in macrophage-rich areas of adventitia, media and intima. Double staining for CD16 and CX3CR1 showed co-localization of these markers, although different patterns were observed (Fig. 5c). In contrast, only few to moderate numbers of CCR2-positive cells (median scores 2–3) were found in these same areas (Fig. 5a,b). Double staining for CD16 and CCR2 confirmed that CD16+ cells rarely co-localize with CCR2 (Fig. 5d).

Next, we analysed tissue expression of chemokines CCL2 and CX3CL1. Both in inflamed and non-inflamed TAB we detected expression of CCL2 and CX3CL1 by VSMCs (Figs 5e and S3). Quantification of CCL2 and CX3CL1 in inflamed temporal arteries demonstrated expression of both chemokine receptor ligands in all three vessel layers with highest expression in the media (Fig. 5e,f). Thus, the phenotype of tissue macrophages in GCA TABs (CD68 + CD16 + CX3CR1 + CCR2−) resembles the phenotype of the non-classical monocytes in blood.

## Discussion

Monocytes/macrophages are critical contributors to inflammatory diseases. Improved understanding of the role of monocyte/macrophages in systemic and local inflammation as seen in GCA and the closely related PMR may provide a rational for novel, prednisone-sparing treatment strategies. Our studies revealed a clear monocytosis in newly diagnosed GCA and PMR patients which is consistent with the notion of inflammation-induced monocyte recruitment from the bone marrow as observed in other inflammatory diseases^22, 23^. When analysing the contribution of the different monocyte subsets to the rise in total blood monocytes, we found numbers of classical monocytes to be substantially increased in both GCA and PMR patients. The increase in classical monocytes led to a relative decrease of non-classical monocytes in these patient groups. Interestingly, glucocorticoid treatment further suppressed non-classical monocytes in GCA and PMR.

The increase of classical monocytes in newly-diagnosed GCA and PMR is likely due to bone marrow production of new monocytes in response to inflammation. Although all three monocyte subsets can be found in the bone marrow^24, 25^, monocyte recruitment from the bone marrow is mainly driven by the CCL2/CCR2 pathway which would explain preferential recruitment of classical monocytes expressing high levels of CCR2^26^. In line with this, we found systemic CCL2 levels decreased in newly diagnosed GCA patients, suggesting binding of this ligand to increased numbers of classical monocytes. Glucocorticoid treatment normalized serum levels of CCL2. A similar pattern was observed in PMR patients.

Although monocytosis is a characteristic of many inflammatory diseases such as RA^22^, Crohn’s disease^23^ and sepsis^27^, several further studies in these indications report on elevated numbers or proportions of CD16+ monocytes^28–30^. In contrast, we did not detect an increase in numbers and proportions of CD16+ monocytes in newly diagnosed GCA and PMR patients. Rather, a proportional decrease of non-classical monocytes particularly, was noted. This is remarkable as classical monocytes are expected to develop into intermediate and non-classical monocytes. The lack of an increase in non-classical monocytes could be due to blunted (cause unknown) differentiation of intermediate monocytes to non-classical monocytes^9, 31^. M-CSF and GM-CSF are thought to be the most potent inducers of differentiation towards the non-classical phenotype^12, 32, 33^. Better understanding of M-CSF and GM-CSF signalling in GCA and PMR patients may elucidate whether differentiation to non-classical monocytes is blunted in GCA and PMR. Alternatively, a selective loss of non-classical monocytes through spontaneous apoptosis, to which the CD16+ monocytes are more susceptible^34^ may underlie the proportional decrease of non-classical monocytes, a notion to be further explored.

Previous studies on the role of non-classical monocytes in homeostasis and in inflammation have revealed that non-classical monocytes actively patrol the vascular endothelium and are preferentially found in the marginal pool^35, 36^. Indeed, non-classical monocytes demonstrate an increased capacity to adhere to endothelial cells by virtue of the adhesion-related CX3CR1 which binds to the membrane-bound form of fractalkine (CX3CL1) expressed by endothelial cells^36, 37^. In the resting state, they clear damaged cells and debris and in infection or inflammation they are thought to be important in resolution of inflammation. Yet, in several disease conditions, non-classical monocytes may aggravate disease, possibly due to their pro-inflammatory potential and following *in situ* reprogramming^8, 28^. More recently, Mukherjee *et al*. showed that untouched non-classical monocytes indeed become pro-inflammatory upon activation^8, 38^. In line with this, non-classical monocytes are potent producers of the pro-inflammatory cytokine IL-6, which is a key molecule in the immunopathogenesis of GCA and PMR^39^. Indeed, elevated levels of IL-6 are found in the serum of newly diagnosed patients^5, 40^. Taken together, the relative decrease of non-classical monocytes in GCA and PMR may be explained by an enhanced accumulation of non-classical monocytes in the marginal pool^35^, thereby facilitating preferential migration of non-classical monocytes to sites of inflammation in GCA and PMR. The latter notion was studied here in GCA TABs only.

We are the first to describe a massive accumulation of CD16+ macrophages in TAB of GCA patients. CD16+ macrophages were found in all layers of the vascular wall. As the adventitia is considered to be the site of primary immunological activation in GCA^41^, this transmural inflammation is likely achieved following entry via the vasa vasorum in the adventitial layer, progressing to the medial and intimal layers of the vascular wall. Accumulation of CX3CR1 + CD16+ macrophages, rarely co-localizing with CCR2, indicates that most of the macrophages in the GCA TAB resemble the phenotype of non-classical monocytes in blood. Still, we cannot exclude the possibility that both CD16 and CX3CR1 expression can be acquired after migration of classical monocytes to tissue. Also, classical monocytes may gain CX3CR1 and lose CCR2 upon migration to tissue^42^. Thus, it remains to be established if non classical monocytes are the only precursors of tissue macrophages in GCA.

Previously, it was proposed that local proliferation of tissue-resident precursors may give rise to macrophage expansion at local sites rather than infiltration by monocytes^43^. We investigated this option but found that staining for the proliferation marker ki-67 was largely negative in TABs of GCA patients (data not shown), thereby excluding this notion.

The strong local expression of CX3CL1 in the GCA TAB is consistent with massive expression of CX3CR1. The presence of CD16+ monocytes in the tissue of GCA may thus be guided by the CX3CR1-CX3CL1 chemokine axis. Although CCL2 is also expressed in lesions of temporal artery tissue, it seems that CCL2 is less important for the migration of CD16+ monocytes. In early *in vitro* migration studies, it was noted that non-classical monocytes failed to migrate in response to CCL2, consistent with the absence of CCR2 on these cells^44^. It was also evidenced that in the absence of CCL2 action, i.e., in CCR2-/- mice, monocytes can still traffic into sites of infection^26^. Importantly, many studies described the importance for chemokine receptor CX3CR1 for tissue migration^19, 45, 46^.

Our study illustrated that glucocorticoid treatment led to clear reductions of non-classical monocyte counts in both GCA and PMR patients. Glucocorticoid treatment has been described to induce apoptosis of non-classical monocytes via a caspase-dependent mechanism^47, 48^. Selective glucocorticoid -induced apoptosis of non-classical monocytes may be explained by high expression of the glucocorticoid receptor in this monocyte subset^47^. Whether glucocorticoids also deplete the CD16+ macrophages in the GCA temporal artery, or in PMR synovial tissues, awaits further studies.

Glucocorticoid treatment led to clear reduction of CX3CR1 per cell expression by peripheral blood monocytes and an up regulation of serum CX3CL1. Thus, glucocorticoid treatment may have a dual effect. Induction of apoptosis on the one hand and reduced adhesion to endothelium by non-classical monocytes through suppression of CX3CR1 expression on the other hand; both effects will hamper the influx of new CD16+ monocytes to the tissue. Interestingly, we found a differential response of monocyte subsets to glucocorticoid treatment in PMR patients. All three subsets were down modulated in PMR patients in remission at 3 months. This is an unexpected finding as the accumulated glucocorticoid dose is substantially lower in PMR patients. An unexplored possibility is that classical monocytes in PMR are more sensitive to glucocorticoid treatment due to their altered characteristics; a previous study showed an altered functionality of monocytes from PMR patients when compared with monocytes from GCA patients and controls^49^.

GCA and PMR are clinically closely related. Evidence for a common immunopathology, however, is lacking due to a paucity of histological data on TAB and synovial tissues obtained from PMR patients. Although our study showed similar changes in circulating monocyte subsets in both diseases, the local involvement of CD16+ monocytes/macrophages in PMR tissues remains to be established.

Other limitations, imposed by logistical constraints, involve the use of thawed PBMCs for the determination of monocyte subset numbers. Whole blood monocyte counts were established using a hematology counter and monocyte subsets were analysed on a later date using thawed samples of liquid nitrogen stored PBMCs (isolated on the day of blood withdrawal). Percentages of monocyte subsets were related to the whole blood monocyte counts to calculate the numbers of the monocyte subsets. We cannot exclude differential loss of cells during PBMC isolation or due to the freezing/thawing procedure, although this would not be expected to differ between patients and controls.

We show an altered monocyte subset distribution in GCA and PMR with a relative decrease of non-classical monocytes. Moreover, macrophages in temporal arteries of GCA patients were found to resemble the phenotype of non-classical monocytes. The data can be taken to suggest that driven by the CX3CL1 chemokine non-classical monocytes infiltrate the arterial wall and develop into an inflammatory population of CD68 + CD16 + CX3CR1 + CCR2− macrophages in GCA. Glucocorticoids reduce the number of non-classical monocytes in blood and their expression of the CX3CR1 receptor, supporting a concept that the influx of new monocytes into the vessel wall is decreased by glucocorticoids leading to the eventual resolution of local inflammation. New insights into the role of monocytes/macrophages in systemic and local inflammation as seen in GCA and the clinically overlapping PMR may provide a rational towards novel treatment strategies and help the identification of highly awaited, disease-specific biomarkers.

## Materials and Methods

### Study populations

Peripheral blood (PB) analysis: in a prospective study design, 42 patients who were newly diagnosed as having GCA (n = 22) or PMR (n = 20) were consecutively enrolled (Tables 1 and S1A,B). None of the patients were receiving glucocorticoids (prednisone) or disease-modifying anti-rheumatic drugs (DMARDs) at the time of blood withdrawal. Blood samples were obtained before noon and all donors were non-fasted. nGCA patients either had a positive TAB and/or positive ^18^F-fluorodeoxyglucose-positron emission tomography-computed tomography (FDG PET-CT). nPMR patients fulfilled the Chuang/Hunder criteria or showed a positive FDG PET-CT scan for PMR^50^. Diagnosis of the PMR patients did not change to GCA during a follow-up period of at least 6 months. As controls, we obtained blood samples from 24 age-matched, healthy controls (HC) who were screened for past or actual morbidities (Tables 1 and S1C).

We obtained 30 follow-up samples of GCA (n = 15) and PMR (n = 15) patients, who were in remission after 3 months of prednisone treatment. Remission was defined as absence of clinical signs and symptoms and a normal erythrocyte sedimentation rate (ESR) (<30 mm/hr) and/ or c-reactive protein (CRP) <5 mg/L.

TAB immunohistochemistry study: TAB were obtained from a total of 16 biopsy positive GCA patients. Eight biopsy positive TAB were included from the 11 TAB available from the PB cohort. In addition, 8 nGCA patients who had a positive TAB were included in the study (Supplementary Table S1A).

Written informed consent was obtained from all study participants. All procedures were in compliance with the declaration of Helsinki. The study was approved by the institutional review board of the UMCG (METc2012/375 for HC and METc2010/222 for GCA and PMR patients).

### Patients treatment

GCA patients were initially treated with 40–60 mg/day (median dose; range 30–60) and PMR patients with 15–20 mg/day (median dose; range 10–40) of prednisone, respectively. Tapering of prednisone treatment was started after 2–4 weeks, based on normalization of clinical signs and symptoms together with normalization of the ESR and/or CRP. After 3 months, the median prednisone dose was 25 mg/day (range 15–50) in GCA patients and 15 mg/day (range 5–17.5) in PMR patients.

### Flowcytometry

Absolute numbers of monocytes in freshly drawn PB samples were determined by BD MultiTest TruCount, as described by the manufacturer. Data were acquired on a FACS Canto-II (BD Biosciences) and analysed with FACSCanto Clinical Software (BD). PB mononuclear cells (PBMC) were isolated from fresh heparinized blood with Lymphoprep (Axis-Shield), frozen in medium with 10% DMSO/FCS, and stored in liquid Nitrogen for analysis at a later date. Thawed PBMCs were stained with the following mAb to quantify monocyte subsets and their chemokine receptor expression: CD3, CD14, CD16, CD19, CD56, CD66b, CCR2 and CX3CR1 (Supplementary Table S3). Proper isotype controls were included. Cells were fixed and analysed using a LSR-II (BD) flowcytometer. Kaluza software (BD) was used for analysis. Classical (CD14^bright^CD16^neg^), intermediate (CD14^bright^CD16^+^) and non-classical (CD14^dim^CD16^+^) monocytes subsets were gated as previously described^51^.

### Immunohistochemistry (IHC)

TAB were obtained from 16 biopsy confirmed GCA patients (Supplementary Table S1A). The tissue was fixed in formalin and paraffin embedded. Tissue sections of 3 μm were deparaffinized and rehydrated. After antigen retrieval and endogenous peroxidase blocking, sections were incubated with anti-human primary antibodies detecting cellular markers CD16, CD68, CD56, CCR2 and CX3CR1 and the chemokines CCL2 and CX3CL1 (product information in Supplementary Table S4). Proper isotype controls were included. Next, slides were incubated with secondary antibody rabbit anti-mouse HRP (DAKO P0260) or goat anti-rabbit HRP (DAKO P0448). Following washing, slides were incubated with peroxidase (DAKO, Carpinteria, CA, USA, P0448 and DAKO P0260). After detection of peroxidase activity with 3-amino-9-ethylcarbazole, slides were counterstained with haematoxylin. Since the temporal artery vessel walls of GCA contain skip lesions, detection of CD16-, CD68-, CD56-, CCR2-, CX3CR1- expressing cells (irrespective of intensity) in affected areas was semi-quantitatively scored on a five-point scale (0–4): 0 = no positive cells, 1 = occasional positive cells (0–1% estimated positive), 2 = low numbers of positive cells (>1–20%), 3 = moderate numbers of positive cells (>20–50%), 4 = high numbers of positive cells (more than 50%). Affected regions containing infiltrating cells were scored. Scoring was performed by two independent investigators, trained by a pathologist and average scores were calculated.

For quantification of tissue chemokine expression, stained sections were scanned using a Nanozoomer Digital Pathology Scanner (NDP Scan U10074–01, Hamamatsu Photonics K.K., Hamamatsu, Japan). Positivity of the staining was quantified (positive pixels/total number of pixels) for representative areas of the three vessel layers (total area >1 × 10^4^ µm^2^) within infiltrated areas using software of Aperio ImageScope (V11.2.0.780 Aperio Technologies, CA, USA).

### Immunofluorescence

To investigate if CD68 and CD16, CD16 and CX3CR1 or CD16 and CCR2 are co-expressed by cells *in situ*, double-labelling immunofluorescence stainings were performed. Formalin-fixed paraffin-embedded TAB tissue was deparaffinized and antigen retrieval was performed. Dilutions (1:50) of anti-CD68, anti-CD16, anti-CCR2, or anti-CX3CR1 antibodies were added and incubated overnight. Following washing, FITC-labelled goat anti-rabbit IgG (A11008, Lifetechnologies, Carlsbad, CA, USA) and AF555-labeled donkey anti-mouse IgG (A31570, Lifetechnologies) were used as the secondary antibodies, respectively. DAPI (10236276001, Roche Life Science, Penzberg, Upper Bavaria, Germany) was performed to stain nuclei. Images were taken using Leica DFC345 FX.

### Serum chemokine measurements

Serum levels of CCL2 and CCL11 (ligands of CCR2) and CX3CL1 and CCL26 (ligands of CX3CR1) were measured by Human premix Magnetic luminex screening assay kit (R&D system, Minneapolis, MN, USA) according to the manufacturers’ instructions. The assay was read by the Luminex LX100™ (Luminex, Austin, TX, USA) multiplex assay detection system. Raw data were analysed using Star Station V2.3. Lower and upper detection limits for the chemokine assays are 3–7936 pg/mL for CCL2, 8–384218 pg/mL for CX3CL1, 2–5963 pg/mL for CCL26 and 8–30043 pg/mL for CCL11.

### Statistical analysis

Since flowcytometry data and serum cytokine data are not normally distributed, non-parametric tests were used for data analysis. To compare data among more than two study groups the non-parametric Kruskal-Wallis test was performed. The Mann-Whitney U test was used to compare data of patient groups with HC. Paired samples (patients at diagnosis and after 3 months of treatment) were compared with the Wilcoxon signed rank test. Analyses were performed with GraphPad Prism 5.0 software. Correlations were assessed using Spearman’s rank correlation coefficient. P-values of less than 0.05 (2-tailed) were considered statistically significant.

### Data availability

The datasets generated during and/or analyed during the current study are available from the corresponding author on reasonable request.

## Electronic supplementary material

Supplementary information
