# Supplementary material for: Involvement of Monocyte Subsets in the Immunopathology of Giant Cell Arteritis
Source: Sci Rep. 2017 Jul 26;7:6553. doi: 10.1038/s41598-017-06826-4 (PMC5529580; doi:10.1038/s41598-017-06826-4)
Supplement: Supplementary file 1 — Supplementary information [file 41598_2017_6826_MOESM1_ESM.pdf]

Supplementary Information page 1-11

Involvement of Monocyte Subsets in the Immunopathology of Giant Cell Arteritis

Yannick van Sleen, Qi Wang, Kornelis S. M. van der Geest, Johanna Westra, Wayel H. Abdulahad, Peter Heeringa, Annemieke M. H. Boots, Elisabeth Brouwer

Table S1A Characteristics of GCA patient cohort and overview of sample inclusion per patient

Patient ID	Sex	Age	Diagnosis	Cell count PB	Monocyte Subsets (%) PB	Chemokine Receptors PB	Chemo-kines Serum	Follow-up Cell count PB	Follow-up %Monocytes Subsets PB	Follow-up Chemokine Receptors PB	Follow-up Chemokines Serum	IHC study	GC before biopsy
GCA7	M	65	PET-CT	X	X		X	X	X	X			
GCA17*	F	81	PET-CT	X	X	X		X	X	X			
GCA13*	M	56	PET-CT	X	X	X			X	X			
GCA2	F	52	PET-CT	X	X	X	X	X	X	X			
GCA10	F	60	PET-CT	X	X	X	X	X	X	X			
GCA9	F	78	PET-CT	X	X	X	X	X	X	X	X		
GCA15	F	69	TAB	X	X	X	X	X	X	X	X		
GCA11	M	79	PET-CT	X	X	X	X	X	X	X	X		
GCA4*	F	68	PET-CT		X	X	X	X	X	X	X		
GCA18	F	62	PET-CT	X	X	X							
GCA16	M	62	TAB	X	X	X	X						
GCA23*	F	76	PET-CT	X	X	X	X						
GCA3	F	68	PET-CT	X	X	X	X						
GCA1	F	79	TAB	X	X	X	X						
GCA8	F	59	PET-CT + TAB	X	X	X	X	X	X	X	X	X	N
GCA12	F	73	TAB	X	X	X	X	X	X	X	X	X	N
GCA6	F	74	PET-CT + TAB	X	X	X	X	X	X	X	X	X	N
GCA14*	F	73	PET-CT + TAB	X	X	X	X	X	X	X		X	N
GCA20	F	79	TAB	X	X	X	X	X	X	X	X	X	N
GCA21	M	77	PET-CT + TAB	X	X	X	X	X	X	X	X	X	N
GCA5	F	73	PET-CT + TAB	X	X	X	X					X	N
GCA22	M	66	PET-CT + TAB	X	X	X	X					X	N
GCA1010	F	76	TAB									X	Y
GCA25	M	60	PET-CT + TAB									X	Y
GCA26	M	65	PET-CT + TAB									X	Y
GCA1001	F	78	TAB									X	Y
GCA1012	F	73	TAB									X	N
GCA1002	M	71	TAB									X	N
GCA000	M	71	TAB									X	N
GCA1009	F	72	TAB									X	N
Figures				1,2, S1A	S1B	4		1,2, S1A	S1B	4		3,5, S2,S3	
Tables							S2				S2	S3, S4	

GCA = Giant cell arteritis. * GCA patients with concomitant PMR. PB: peripheral blood; M: male; F: Female; PET-CT: positron emission tomography-computer tomography; TAB: temporal artery biopsy; GC: glucocorticoids; X: sample was included; N: no GC therapy; Y: GC treatment started before biopsy was taken.

Table S1B. PMR patient cohort and overview of sample inclusion per patient

Patient ID	Sex	Age	Diagnosis	Cell count PB	Monocyte Subsets (%) PB	Chemokine Receptors PB	Chemokines Serum	Follow-up Cell count PB	Follow-up %Monocytes Subsets PB	Follow-up Chemokine Receptors PB	Follow-up Chemokines Serum
PMR501	M	62	Chuang + PET-CT	X	X	X	X	X	X	X	X
PMR504	M	58	Chuang	X	X	X	X	X	X	X	X
PMR505	F	74	Chuang + PET-CT	X	X	X	X	X	X	X	X
PMR512	F	81	Chuang + PET-CT	X	X	X	X	X	X	X	X
PMR518	F	64	Chuang + PET-CT	X	X	X	X	X	X	X	X
PMR515	F	77	Chuang + PET-CT	X	X	X	X	X	X	X	X
PMR517	F	79	Chuang	X	X	X	X	X	X	X	X
PMR517	F	79	Chuang	X	X	X	X	X	X	X	X
PMR502	F	75	Chuang + PET-CT	X	X	X		X	X	X	
PMR503	M	82	PET-CT	X	X	X	X	X	X	X	
PMR516	F	75	Chuang + PET-CT	X	X	X	X	X	X	X	
PMR513	F	76	Chuang	X	X	X	X	X	X	X	
PMR520	F	65	Chuang + PET-CT	X	X	X	X	X	X	X	
PMR514	M	54	Chuang + PET-CT	X	X	X	X	X	X		X
PMR507	F	68	Chuang + PET-CT		X	X	X	X	X	X	X
PMR000	F	61	Chuang	X	X	X	X				
PMR509	F	69	Chuang + PET-CT	X	X	X	X				
PMR511	M	84	Chuang	X	X	X	X				
PMR506	M	82	Chuang + PET-CT	X	X	X	X				
PMR510	F	62	Chuang + PET-CT	X	X	X					
Figures				1,2, S1A	S1B	4		1,2, S1A	S1B	4	
Tables							S2				S2

PB: peripheral blood; M: male; F: Female; PET-CT: positron emission tomography-computed tomography; TAB: temporal artery biopsy; GC: glucocorticoids; X: sample was included.

Table S1C. HC cohort and overview of sample inclusion per donor

ID	Sex	Age	Cell count PB	% Monocyte Subsets PB	Chemokine Serum
SEN5	M	65	X	X	X
SEN14	F	75	X	X	X
SEN501	F	53	X	X	X
SEN55	F	67	X	X	X
SEN57	F	66	X	X	X
SEN75	F	75	X	X	X
SEN79	F	79	X	X	X
SEN61	F	72	X	X	X
SEN66	M	72	X	X	X
SEN62	M	73	X	X	X
SEN3	F	71	X	X	X
SEN6	F	62	X	X	X
SEN58	F	62	X	X	X
SEN10	M	71	X	X	X
SEN32	M	80	X	X	X
SEN39	F	73	X	X	X
SEN47	F	63	X	X	X
SEN59	M	61	X	X	
SEN60	F	59	X	X	
SEN30	F	64	X	X	
SEN54	F	74		X	X
SEN25	F	75		X	
SEN26	F	79		X	
SEN45	F	83		X	
Figures			1,2, S1A	S1B	
Tables					S2

HC: Healthy control; PB: peripheral blood; M: male; F: Female; X: sample was included.

Table S2. Serum chemokine concentrations in healthy controls, GCA and PMR patients before and after glucocorticoid treatment

pg/mL	HC	nGCA	rGCA	nPMR	rPMR
CCL2	409<br>(187-589)	281<br>(42-595)<br>*p= 0.032**	400<br>(221-893)<br>*p=0.0078^*	322<br>(172-673)	475<br>(146-725)
CCL11	114<br>(68-201)	68<br>(30-356)<br>*p=0.0057**	178<br>(127-873)<br>*p=0.0078^*	99<br>(50-178)	140<br>(68-277)
CX3CL1	956<br>(728-1789)	924<br>(526-1969)	1177<br>(794-19175)<br>*p=0.0391^*	891<br>(594-1789)	956<br>(661-2265)
CCL26	4<br>(4-48)	4<br>(4-109)	10<br>(4-29)	4<br>(4-10)	4<br>(4-10)

Systemic concentrations of CCL2 and CCL11 (CCR2 receptor ligands) and CX3CL1 and CCL26 (CX3CR1 ligands) were measured in healthy controls (HC, n=18), newly-diagnosed patients with GCA (nGCA; n=19) and PMR (nPMR; n=18) and in the follow-up samples of GCA (rGCA; n=9) and PMR (rPMR; n=10) patients in remission after 3 months of glucocorticoid treatment. Results are expressed as median values and range in pg/mL. The Kruskal-Wallis test was performed to compare data among study groups. The Mann-Whitney U test was used to compare each patient group with HC and is indicated with *. Paired samples rGCA vs nGCA and rPMR vs nPMR) were compared with the Wilcoxon signed rank test and is indicated with ^. P-values of less than 0.05 (2-tailed) were considered statistically significant.

Table S3. Primary Antibody information used in Flow Cytometry

Antibody	Conjugation	Clone	Supplier
**Anti-CD2**	PE-Cy7	RPA-2.10	eBioscience, San Diego, CA, USA
**Anti-CD3**	APC	UCHT1	BD Biosciences Franklin Lakes, NJ, USA
**Anti-CD3**	EF605	OKT3	eBioscience
**Anti-CD14**	PE	M5E2	Biolegend, San Diego, CA, USA
**Anti-CD16**	V450	3G8	BD Biosciences
**Anti-CD16**	AF700	3G8	Biolegend
**Anti-CD19**	APC-eFluor780	HIB19	eBioscience
**Anti-CD19**	EF605	HIB19	eBioscience
**Anti-CD56**	BV510	HCD56	Biolegend
**Anti-CD56**	FITC	MEM188	eBioscience
**Anti-CD66b**	PE-Cy7	G10F5	eBioscience
**Anti-CCR2**	PerCP-Cy5.5	K036C2	Biolegend
**Anti-CX3CR1**	FITC	2A9-1	Biolegend
**Anti-IL-6**	APC	MQ2-13A5	eBioscience

Table S4. Primary Antibody information used in Immunohistochemistry

Antibody	Isotype	Clone	Supplier / Cat #	Dilution	Antigen retrieval
**Anti-CD16**	Rabbit IgG	SP175	Abcam (Cambridge, UK) ab183354	1:50	10mM tris-HCL+ 1mM EDTA pH=9
**Anti-CD68**	Mouse IgG3κ	PG-M1	DAKO (Troy, Mich, USA), M0876	1:50	idem
**Anti-CD56**	Mouse IgG2b	MEM-188	Abcam, ab8233	1:100	idem
**Anti-CCR2**	Mouse IgG2a	7A7	Abcam, ab176390	1:50	idem
**Anti-CX3CR1**	Mouse IgG1	8E10.D9	Biolegend, 824001	1:50	idem
**Anti-CCL2**	Mouse IgG2b	23002	R&D (Minneapolis, Minn, USA), MAB679	1:5	idem
**Anti-CX3CL1**	Rabbit IgG#,	NA	Abcam, ab25088	1:100	idem

protein A purified polyclonal antibodies. NA = not applicable

Legends to the Supplementary Figures

Figure S1. Cumulative schematic overview of the three monocyte subsets in PBMC from GCA/PMR. **A**, A cumulative schematic overview of the median values of absolute numbers of classical, intermediate, and non-classical monocytes in healthy controls (HCs, n=20), newly-diagnosed patients with GCA (nGCA; n=21) and PMR (nPMR; n=19) and in the follow-up samples of GCA (rGCA; n=14) and PMR (rPMR; n=15) patients in remission after 3 months of prednisone treatment. **B**, A cumulative schematic overview of the proportions (percentages of total monocytes) of classical, intermediate, and non-classical monocytes in the study groups. The Kruskal-Wallis test was performed to compare data among study groups (HCs, GCA and PMR). The Mann-Whitney U test was used to compare nGCA and nPMR with HCs and is indicated with *. Paired samples were compared with the Wilcoxon signed rank test and is indicated with ^.

Figure S2. Isotype controls for immunohistochemical staining.

Immunohistochemical staining for isotype controls e.g. mouse IgG1, mouse IgG2a, mouse IgG2b, Rabbit IgG in a representative inflamed temporal artery biopsy specimen from GCA patients using equivalent antibody concentrations.

Figure S3. Comparison immunohistochemical staining of non-inflamed and inflamed temporal artery biopsies from GCA patients.

Left panels show non-inflamed TABs and right panels show GCA TABs. Representative staining for CCR2, CX3CR1, CCL2 or CX3CL1 is shown using equivalent antibody concentrations. Non-inflamed TABs lack infiltrates and tissue remodeling typical of GCA and consist mainly of vascular smooth muscle cells.

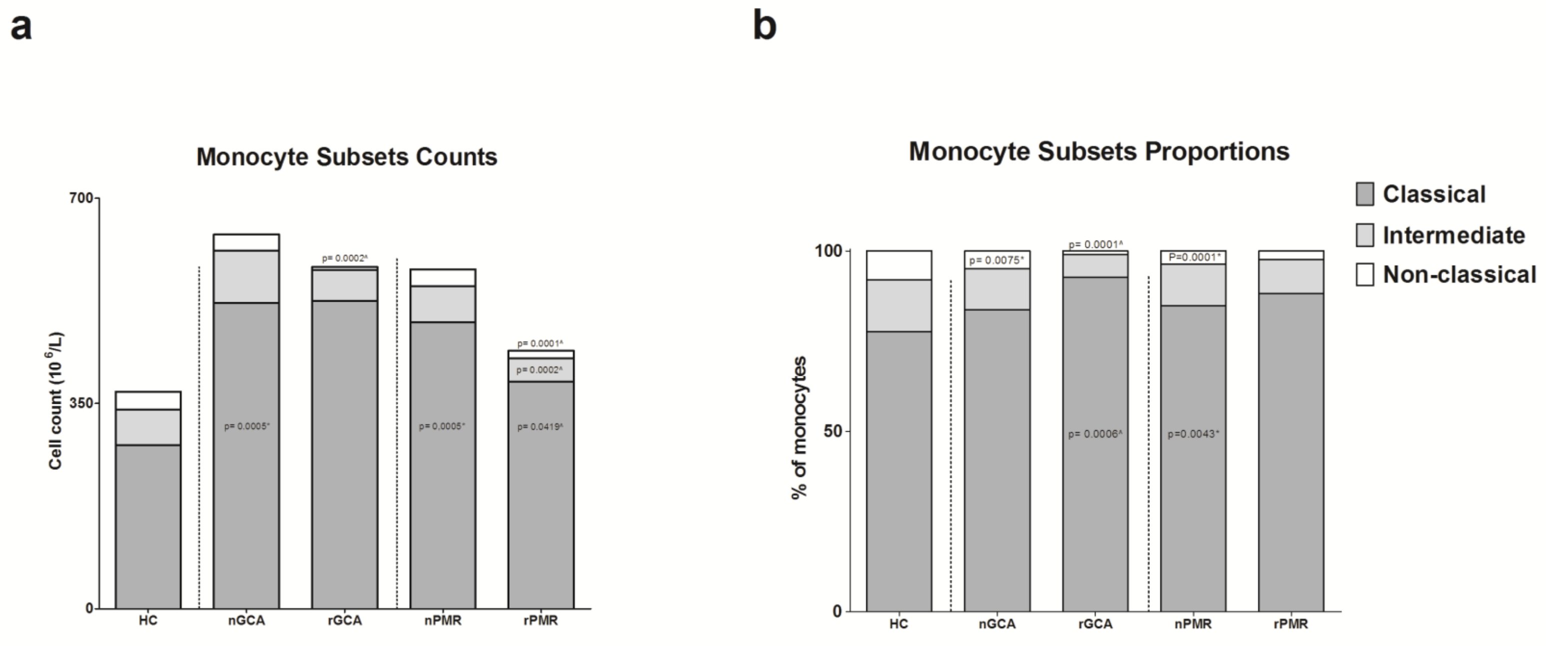
a
Monocyte Subsets Counts
Cell count (10^6/L)
700
350
0
HC
nGCA
rGCA
nPMR
rPMR
p= 0.0005*
p= 0.0002^
p= 0.0005*
p= 0.0001^
p= 0.0002^
p= 0.0419^
b
Monocyte Subsets Proportions
Classical
Intermediate
Non-classical
% of monocytes
100
50
0
HC
nGCA
rGCA
nPMR
rPMR
p= 0.0075*
p= 0.0001^
P=0.0001*
p= 0.0006^
p=0.0043*

Figure S1.

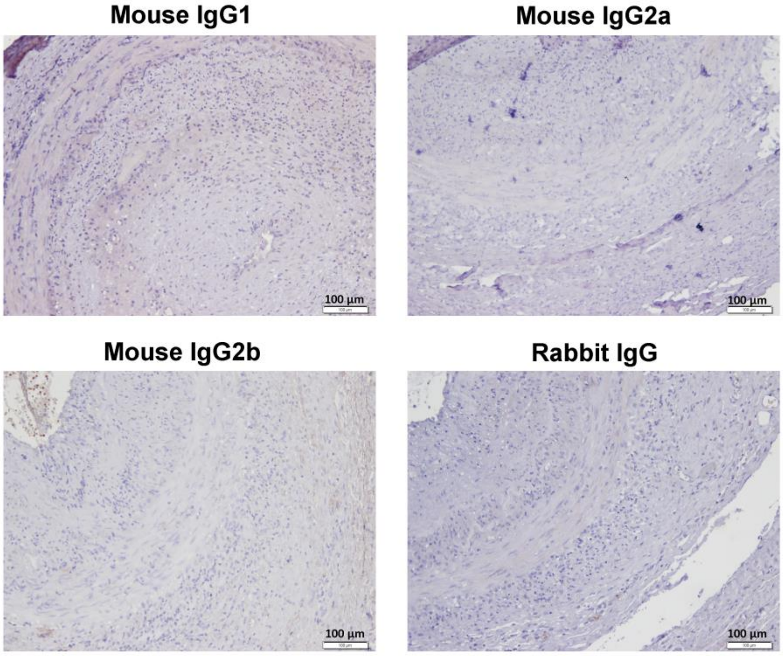
Mouse IgG1
Mouse IgG2a
Mouse IgG2b
Rabbit IgG
100 µm
100 µm
100 µm
100 µm

Figure S2.

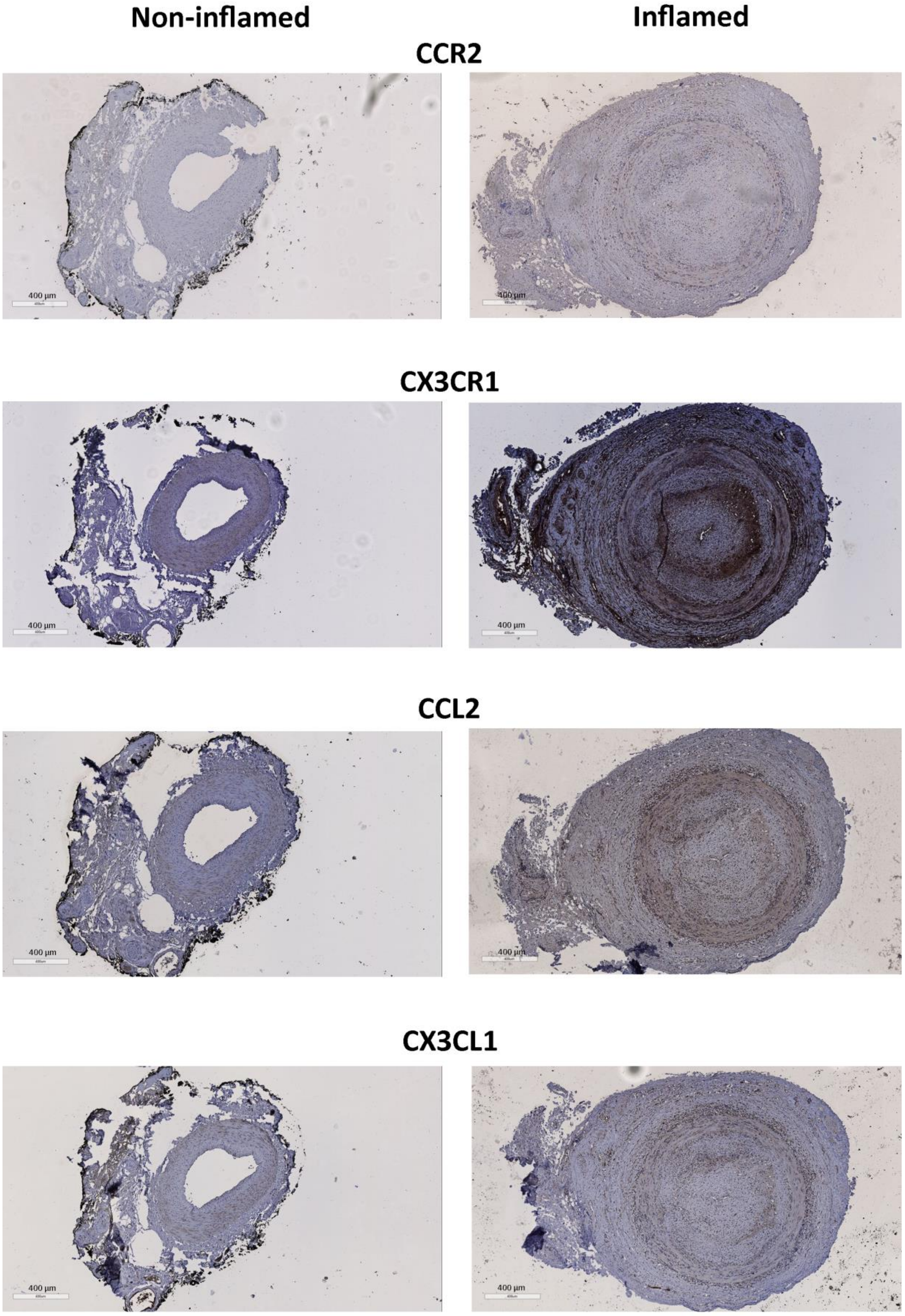

Figure S3
